# Research Progress on the Mechanism of the Impact of Myofibrillar Protein Oxidation on the Flavor of Meat Products

**DOI:** 10.3390/foods13203268

**Published:** 2024-10-15

**Authors:** Lingping Zhang, Dongsong Yang, Ruiming Luo, Yulong Luo, Yanru Hou

**Affiliations:** 1 College of Food Science and Engineering, Ningxia University, Yinchuan 750021, China; 2 Department of Health and Wellness Service Industry, Guangzhou Light Industry Technician College, Guangzhou 510220, China

**Keywords:** protein, myofibrillar protein, oxidation mechanism, flavor, antioxidant, regulate

## Abstract

Myofibrillar proteins primarily consist of myosin, actin, myogenin, and actomyosin. These proteins form complex networks within muscle fibers and are crucial to the physical and chemical properties of meat. Additionally, myofibrillar proteins serve as significant substrates for the adsorption of volatile flavor compounds, including aldehydes, alcohols, ketones, and sulfur and nitrogen compounds, which contribute to the overall flavor profile of meat products. A series of chemical reactions occur during the processing, storage, and transportation of meat products. Oxidation is one of the most significant reactions. Oxidative modification can alter the physical and chemical properties of proteins, ultimately impacting the sensory quality of meat products, including flavor, taste, and color. In recent years, considerable attention has been focused on the effects of protein oxidation on meat quality and its regulation. This study investigates the impact of myofibrillar protein oxidation on the sensory attributes of meat products by analyzing the oxidation processes and the factors that initiate myofibrillar protein oxidation. Additionally, it explores the control of myofibrillar protein oxidation and its implications on the sensory properties of meat products, providing theoretical insights relevant to meat processing methods and quality control procedures.

## 1. Introduction

Meat products, characterized by their elevated fat and protein content, are influenced by various external environmental factors, including temperature, light, oxygen, and moisture, during the stages of processing, storage, and transportation until they are ultimately presented to the consumer. This leads to their deterioration. Multiple research studies have shown that a key element leading to the deterioration in the quality of meat items is oxidation, encompassing protein oxidation and lipid oxidation. Findings indicate that moderate levels of oxidation can enhance the juiciness, flavor, and tenderness of meat products through the production of aldehydes, ketones, volatile fatty acids, and other distinctive flavor compounds [1]. Excessive oxidation of meat products can result in the emergence of unpleasant smells and potentially harmful by-products, which can diminish water retention, tenderness, color, flavor, and nutritional quality. Protein oxidation shares similarities with lipid oxidation in this context.

Meat tissue contains a high concentration of protein, which plays a critical role in the structure of meat tissue, impacting the nutritional, sensory, and physicochemical properties of meat and its by-products. While lipid oxidation in food systems has been extensively researched over time, the impacts and processes of protein oxidation in food, particularly in meat products, have remained largely unexplored.

Protein oxidation in meat is thought to occur during the formation of protein-free radicals. Proteins are induced by reactive oxygen species (ROS) to undergo covalent modification, which occurs through chain reactions of free radicals [2]. Free radicals act on proteins at both the oxo-acid backbone and side chains, often leading to polymerization and fragmentation of the protein [3]. Proteins can undergo oxidation through a variety of pathways, including amino acid oxidation, the establishment of connections with lipid peroxidation by-products, metal-catalyzed oxidation, and oxidation-induced cleavage. Among these mechanisms, metal-catalyzed protein oxidation is notably prominent [4]. Various forms of oxidative harm to proteins in meat, even meat products, can include alterations in structure, fragmentation of peptide chains, and the creation of amino acid by-products or clusters, resulting in modifications to the physicochemical characteristics of proteins and subsequently impacting their functional attributes [5]. Protein oxidation is an important factor that affects flavor. Protein oxidation in meat often occurs to varying degrees during processing and storage. This mechanism leads to the myofibrillar proteins undergoing folding, stretching, and aggregation, which in turn influences the generation of flavor compounds and the interplay between proteins and flavor compounds.

Myofibrillar protein plays a vital role as a key constituent in meat, rich in essential amino acids. The process of oxidation is significantly influential in the assessment of the quality and taste attributes of meat products, as it can alter the flavor profile through the modification of myofibrillar proteins. Hence, it is essential to regulate levels to preserve the quality and flavor of meat products in the course of processing and storage operations.

Therefore, this article reviews meat products and discusses the oxidation mechanism of proteins, oxidation induction mechanism, myoglobin oxidation, and the effect of oxidation regulation on meat product flavor. The aim is to demonstrate the contribution of protein oxidation to meat product flavor. These studies help us become better acquainted with and over the quality and flavor of meat products, thereby promoting the development of the meat industry.

## 2. Myofibrillar Protein Oxidation

### 2.1. The Oxidation Mechanism of Myofibrillar Proteins

**Figure 1:**
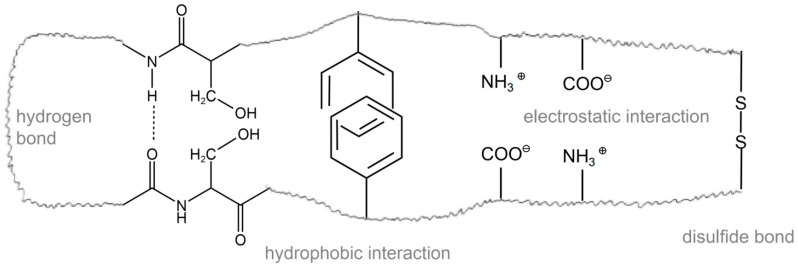
Schematic representation of forces in protein structure.

**Figure 2:**
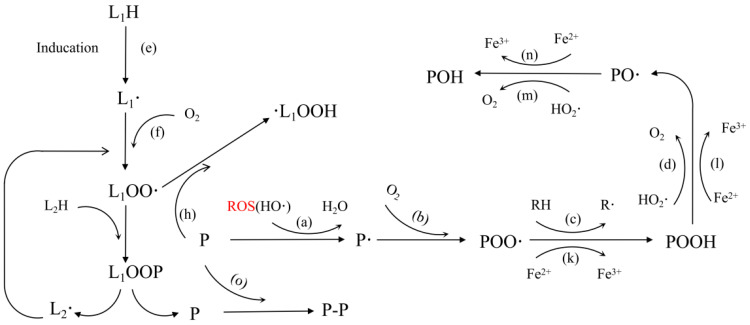
Schematic diagram of myofibrillar protein oxidation mechanism.

The oxidation of myofibrillar proteins is primarily facilitated by reactive oxygen species, including superoxide anions (O_2_^−^) and hydroxyl radicals (·OH). Additionally, this process can be indirectly initiated by oxidation by-products like malondialdehyde (MDA), hydroxynonanal, and acrolein. These oxidants can attack the functional groups of amino acids and the backbone of peptides, resulting in protein degradation, aggregation, and polymerization [6]. First, amino acids containing sulfhydryl (SH) in myofibrillar proteins are covalent bonding sites that are highly susceptible to free radicals. Superoxide anions can react with SH to form disulfides (SS-) and release hydrogen ions (H^+^). This process consumes superoxide anions and produces hydrogen peroxide (H_2_O_2_). Hydrogen peroxide can further decompose into hydroxyl radicals and hydroxide ions (OH-), thereby triggering stronger oxidation reactions [7,8,9]. Hydroxyl radicals can react with other groups like sulfhydryl, amino (NH_2_), carboxyl (COOH), and so on in myofibrillar proteins, resulting in covalent bond breaking and cross-linking within the protein molecule (Figure 1 and Figure 2).

In addition to breaking covalent bonds, myofibrillar protein oxidation also involves noncovalent bond interactions. During oxidation, myofibrillar protein molecules undergo conformational changes, which result in alterations in intermolecular interactions. The exposure or exposure enhancement of groups such as carbonyl (C=O) and lactone (C-CO-C) in myofibrillar protein molecules occurs during oxidation (Figure 1) [10]. These carbonyl and lactone groups can engage in noncovalent bond interactions with neighboring molecules, such as hydrogen bonds, hydrophobic interactions, and others, thereby altering the secondary structure of the protein. In addition to the covalent and noncovalent interactions mentioned above, other mechanisms may take part in the oxidation of myofibrillar proteins. For example, the enzyme-mediated pathway plays a role in the process of protein oxidation. Myofibrillar proteins are hydrolyzed by proteases, resulting in free radical production, subsequent reactions, and cleavage and degradation of protein molecules. This hydrolysis may be related to conformational changes and molecular interactions during oxidation [3].

### 2.2. Mechanism of Protein Oxidation Induction

Protein oxidation is a chemical alteration caused by ROS either through direct interaction or through the activation of specific inducers [11]. Protein oxidation resulting from ROS can induce alterations to both the protein backbone and side chains. These modifications can impact the primary, secondary, and tertiary structures of proteins, thereby influencing their functional characteristics and ultimately impacting the quality properties of meat, like color and flavor [12]. ROS extracts hydrogen atoms from proteins to form carbon-centered radicals, which is the beginning of protein oxidation (Reaction (a)) [13]. Then, in the existence of oxygen, peroxyalkyl radicals are formed (Reaction (b)). Subsequently, this peroxyalkyl radicals capture hydrogen atoms from external molecules (Reaction (c)) to generate alkyl peroxides. It is then converted to the alkoxy radical by the reaction with ROS (Reaction (d)) [14].

P + HO**·** → P**·** + H_2_O (a)

P**·** + O_2_ → POO (b)

POO**·** + P → POOH + P (c)

POOH + HO_2_**·** → PO**·** + O_2_ + H_2_O (d)

Note: P = protein; P**·** = protein carbon-centered radical; POO**·** = protein peroxyalkyl radical; POOH = protein alkyl peroxide; PO**·** = protein alkoxy radical.

#### 2.2.1. Direct Induction of Oxidation Reaction by Free Radicals

Free radical inducers all contain a lone electron. When a protein is deprived of a hydrogen atom by ROS, the oxidation begins. In addition, ROS can also directly act on proteins through oxygenation, coupling, and cleavage reactions. ROS attacks proteins principally at three action sites: the main peptide chain skeleton, side chain groups of aliphatic amino acids, and aromatic amino acids [15,16]. Free radicals convert the carbon-centered group on the backbone of the peptide into alkoxide radical (COO**·**) and alkoxide radical (CO**·**) through hydrogen capture, single-electron reduction, and oxygenation. This procedure has the potential to induce cleavage of the peptide backbone or the creation of cross-links via α-amidation or diamide routes. The alkyl side chains of aliphatic amino acids can be readily oxidized to form carbonyl compounds. In the case of aromatic amino acids, when their side chain groups are targeted by free radicals, the resulting reaction products are transformed into aromatic radicals and aromatic derivatives through coupling reactions and hydrogenation [14,17].

#### 2.2.2. Lipid Oxidation Induces Oxidative Reactions

Lipid oxidation and protein oxidation influence each other, and free radicals and aldehyde groups produced by lipid peroxidation may covalently bind to residues in proteins [1]. During lipid peroxidation, a large number of intermediates, such as COO**·**, CO**·**, reactive carbonyl compounds, and hydroperoxides, are generated. These intermediates can covalently bind with proteins (Reaction (h)) [14], giving rise to subsequent protein aggregation and cross-linking (Reaction (i)). Alternatively, lipid-free radicals can interact with proteins to generate protein-free radicals (Reaction (g)), followed by a protein polymerization reaction (Reaction (j)). Derived by-products of lipid peroxidation that react with nucleophiles in proteins are mainly α- and β-unsaturated aldehydes (acrolein, 4-hydroxy-2-nonenal, and malondialdehyde) [18].

Table 1 lists some possible mechanisms by which free radicals generated by lipid oxidation trigger the production of free radicals in proteins, the formation of polymers, and the formation of protein–lipid complexes.

#### 2.2.3. Metal Ions Induce Oxidation Reactions

Protein oxidation also may be initiated through interactions with metal ions, such as free iron ions that accumulate in muscle tissue following the disruption of internal tissue structure in meat products post-slaughter. This leads to the Fenton reaction occurring with the accumulated hydrogen peroxide in the metabolic process, resulting in an excess of free radicals, which facilitates the oxidative denaturation of proteins [19]. Metal ions like Cu^2+^ and Fe^2+/3+^ can cause oxidation of the sulfhydryl or amino side groups found in amino acids like Cys, His, and Met [20]. Metal ions typically speed up the production of reactive oxygen species, which then capture a hydrogen atom on the protein molecule and create a carbon-centered radical (P**·**) (Reaction (a)), which is modified to POO**·** under aerobic conditions (Reaction (b)). POO**·** can easily undergo redox reactions with Fe^2+^ (Reaction (m)), capture an external hydrogen atom (Reaction (c)), or be attacked by protonated superoxide radicals (Reaction (d)), eventually forming alkyl peroxides (POOH). The reaction of POOH with Fe^2+^ or peroxide radical (HO_2_**·**) can generate alkoxy radical (PO**·**) (Reactions (d) and (l)) [21,22]. Either Fe^2+^ or HO_2_**·** can react with alkoxy radicals to form hydroxyl derivatives (POH) (reactions m and n). Under aerobic conditions, the two P**·** molecules react with each other to form a cross-linked derivative by creating a carbon–carbon chain (P-P) (Reaction (o)).

Fe^2+^ + POO**·** → POOH (k)

POOH + Fe^2+^ → PO**·** + Fe^3+^ (l)

PO**·** + HO_2_**·** → POH + O_2_ (m)

PO**·** + Fe^2+^ → POH + Fe^3+^ (n)

PH + PH → P-P (o)

### 2.3. Oxidation Indices of Proteins

Myofibrillar protein oxidation indices, such as alterations in amino acid residue side chains, carbonyl formation, loss of thiol groups, and the formation of protein cross-links, are crucial for understanding and regulating the quality and flavor of meat products. Modifications in the side chains of amino acid residues directly reflect protein oxidation. These changes may alter the content and proportions of existing flavor substances and affect the protein’s ability to adsorb flavor substances, thereby impacting the overall flavor profile of meat products [23]. The increase in carbonyl groups and the loss of sulfhydryl groups are significant indicators of protein oxidation, reflecting the extent of protein oxidation and alterations in protein structure. These changes can influence the interaction and binding capacity between proteins and flavor compounds. Furthermore, the oxidation of sulfhydryl groups may lead to the production of specific flavor compounds, such as sulfur-containing substances, which significantly impact the flavor of meat products. The formation of protein cross-links can alter the manner in which proteins interact with flavor compounds, thereby affecting the overall flavor profile of meat products. Additionally, the development of cross-linked structures may modify the protein network architecture, influencing the texture and taste of meat products and, consequently, indirectly affecting their flavor.

#### 2.3.1. Side Chain Changes of Amino Acid Residues

Myofibrillar protein is an important structural protein in muscles, and its amino acid side chains contain several essential sulfur compounds, including Cys, Met, and Cys-ss-X. In theory, almost all amino acid side chains in proteins can be modified by ROS. But in reality, different amino acids exhibit varying sensitivities to ROS [13,14]. Certain amino acids such as Tyr, Cys, Met, Trp, Lys, Arg, His, Pro, and Phe are highly sensitive to ROS. Of these, cysteine is the most vulnerable and is typically the first to undergo oxidation. Methionine can also be easily oxidized to create methionine sulfoxide derivatives, while other amino acids may need more severe oxidation conditions [24]. Table 2 shows the amino acid residues and their oxidation products [25].

Apart from sulfur-containing components, the aromatic functional groups found in amino acids are also highly susceptible to protein oxidation. Specifically, tyrosine possesses redox-active properties, with its phenolic side chains being susceptible to oxidation due to the stabilization of intermediate tyrosyl radicals through the dispersion of an unpaired electron mediator [26]. Hydroxyl radicals can rapidly oxidize amino acid side chains, targeting sulfur-containing residues and aromatic functional groups. When hydroxyl radicals oxidize amino acid side chains, the oxidation sequence is as follows: Cys is oxidized to the highest extent, followed by Trp, Tyr, Met, Phe, His, Ile, Leu, and Pro [27].

Modifications to the side chains of amino acid residues can influence the tertiary structure of proteins, thus impacting the binding capacity and stability of proteins to flavor substances, consequently influencing the flavor of meat products. During heat treatment, myosin shows a reduction in α-helical structure and an increase in β-turn, β-sheet, and random coil structures. These changes in secondary structure lead to the unveiling of sulfhydryl groups and active amino acids, thereby generating additional sites for binding [28]. The flavor characteristics of amino acids are intricately linked to the hydrophobic nature of their side chain R groups, with a specific emphasis on factors such as chain length and chain and the abundance of hydrophobic amino acid residues. When the hydrophobicity of amino acids is low, they are generally considered to be sweet, such as glycine, alanine, serine, etc. When the hydrophobicity is high, the taste is mainly bitter, as seen in compounds like Leu, Ile, Phe, etc. [29]. The taste characteristics of amino acids are closely associated with the hydrophobic properties of their side chain R groups, particularly focusing on variables such as the length of the chain and the abundance of hydrophobic amino acid residues.

#### 2.3.2. Generation of Carbonyl

A significant alteration in protein oxidation takes place as a result of the creation of carbonyl groups, and the extent of protein oxidation is typically evaluated by quantifying the amount of carbonyl groups present [30]. Protein carbonylation represents a type of oxidative injury that is caused by oxidative stress and is irreversible. This process often leads to permanent harm to protein configurations as a result of oxidative alterations to amino acid residues located within the protein side chains [31]. Protein carbonylation is a persistent alteration caused by ROS through three mechanisms: direct oxidation of amino acid side chains, fragmentation, and non-enzymatic glycosylation of protein peptide chain backbones (i.e., carbonylation) or fat oxidation [26]. The process of oxidizing the side chains of amino acid residues, including proline, arginine, threonine, and lysine, can lead to the formation of carbonyl derivatives, as demonstrated in Table 1. The deamination reaction, in particular, is likely to be the major process for the formation of protein carbonyl groups. Protein oxidation produces carbonyl compounds, such as alpha-amino adipic acid and gamma-glutamate half aldehyde. Strecker aldehydes, which are common in meat products, especially in dry-salted meat, are volatile components that contribute to the flavor profile [32]. As the duration of oxidation progresses, there will be a gradual rise in the levels of volatile flavor compounds, including aldehydes and ketones [33]. Proteins may interact with volatile aldehydes, leading to the formation of Schiff bases through condensation reactions, wherein the aldehydes react with free amino groups or amino acids of proteins [34].

#### 2.3.3. Loss of Sulfhydryl Groups

A decrease in sulfhydryl content can be used as a marker for protein oxidation, with a greater degree of oxidation resulting in a decline in SH content. The oxidation of SH groups on cysteine residues represents a general measure of various protein modifications [35]. The process of sulfur-hydrogen (SH) oxidation is a multifaceted reaction that can be triggered by different oxidizing agents, such as hydrogen peroxide (H_2_O_2_) and other peroxides. This leads to the generation of a wide array of oxidation by-products, including mixed disulfides (e.g., protein glutathione), cystine (disulfide), nitroso adducts (RS-NO), and various oxicacids (e.g., RSOOH, RSOH, sulfinic acid, RSO_3_H, and RSO_2_H) [36]. The existence of various oxidation products alters the original protein structure, leading to a change in the protein’s initial properties. Reduced thiol can lead to ROS-mediated formation of disulfide bonds in proteins. Some sulfur can covalently bind with the flavor compounds, so reduced sulfur content may influence the combination of protein and flavor substances, affecting the ability to form disulfide bonds and indirectly impacting meat flavor [34].

#### 2.3.4. Formation of Protein Cross-Linking

Cross-linking is a notable process of protein oxidation that entails the creation of covalent bonds between polypeptide chains within a protein (intramolecular cross-linking) or among distinct protein molecules (intermolecular cross-linking). The resulting intra- and intermolecular cross-links in oxidized proteins induce structural alterations that can impair their functionality. The process of intramolecular and intermolecular cross-linking in muscle proteins includes the generation of various cross-linked oxidation products and the subsequent polymerization of proteins [37]. The common protein cross-links are mainly disulfide cross-linking and tyrosine cross-linking, which can occur within molecules or chains. Some of these cross-links play a vital role in maintaining or stabilizing the structure of proteins [38]. Based on existing scholarly sources, it is understood that myofibrillar protein oxidation generates the creation of protein polymers primarily through specific mechanisms: (1) Irreversible carbonylation. Myofibrillar proteins undergo irreversible carbonylation reactions through metal-catalyzed oxidation of residues like lysine, arginine, proline, and threonine, thereby affecting the secondary and tertiary structures of proteins, enhancing protein–protein interactions, and promoting the formation of proteinaceous polymers [39]. (2) Protein phosphorylation is a prevalent and significant post-translational modification that primarily targets tyrosine, threonine, and serine residues, playing an indispensable role in postmortem muscle properties. It affects protein stability and solubility, promoting protein aggregation [40]. (3) Oxidative stress can lead to glycosylation modification of myofibrillar proteins, thereby affecting the aggregation state of proteins and promoting their aggregation. Research has indicated that lysine and arginine residues serve as the primary sites where advanced glycation end products (AGEs) are formed within proteins [41]. In experiments conducted both in living organisms and in controlled laboratory settings, An investigated the impact of varying degrees of cross-linking on the release concentration and rate of aroma compounds. An observed pattern where the concentration and rate of release initially rose and then declined as the cross-linking degree increased. This occurrence is probably attributed to the modification of protein structure, conformation, and physical and chemical properties induced by the cross-linking effect. Consequently, the interaction between proteins and flavor compounds is influenced [42].

## 3. The Effect of Oxidation on the Flavor of Meat Products

In general, meat contains a diverse array of volatile flavor compounds like ketones, aldehydes, alkanes, pyridines, esters, alcohols, acids, pyrazines, sulfur-containing compounds, and more. The generation of flavors in meat products is influenced by the involvement of muscle proteins in flavor formation, primarily through two key mechanisms. First, through protein degradation, free amino acids (FAAs) and small peptides serve as precursors for the formation of flavor compounds. The emission and breakdown of these substances have the potential to control the makeup of volatile and non-volatile compounds, consequently impacting the overall characteristics of meat products [43]. Keska and Stadnik [44] found that myofibrillar proteins, particularly myosin-2, serve as effective precursors for taste-active peptides and amino acids, giving rise to compounds characterized by unique bitter, fresh, and sour taste profiles. This was determined through computational simulation analysis of amino acids present and taste-active peptides in pork. But this effect is very limited. In most cases, proteins can adsorb volatile components by physical or chemical means. Changes in the spatial structure, surface hydrophobicity, and gel characteristics of proteins will change the overall flavor balance to different extents, significantly influencing flavor release [45].

The degree of hydrophobicity exhibited on the surface of a protein serves as a significant indicator of its structural stability. Li et al. [46] showed that when myofibrillar proteins unfold, non-polar amino acids that are normally located within the protein structure become exposed, leading to the creation of a hydrophobic core. This exposure on the protein surface enhances the hydrophobic properties of the protein. Cao et al. [47] investigated the effect of oxidative modification of actin on its interaction with flavor compounds (mainly alcohols and aldehydes) by establishing an H_2_O_2_ oxidation system. They showed that low oxidation treatment (0–5 mmol/L H_2_O_2_) of G-actin can increase surface hydrophobicity and carbonyl content, reduce sulfhydryl, and enhance alcohol binding and aldehyde release through hydrogen bonding. On the contrary, high oxidation treatment (5–20 mmol/L H_2_O_2_) of G-actin resulted in an increase in the hydrophobicity, which increased the absorption of aldehydes.

Muscle proteins play a vital role in the modulation of taste and overall characteristics of meat products. Myofibrillar proteins, which consist of tropomyosin, myosin, and actin, are the primary constituents of muscle proteins. The appearance, flavor, and texture properties of meat are dramatically influenced by the intramolecular and intermolecular interactions of myofibrillar proteins, which involve various types of bonds, including hydrogen, hydrophobic, ionic, and van der Waals forces [48]. Cao et al. [47] utilized scanning electron microscopy and gas chromatography-mass spectrometry (GC-MS) techniques to demonstrate that the clustered G-actin promotes hydrophobic interactions with aromatic compounds, resulting in the creation of complexes between proteins and aromatic compounds, thereby augmenting their binding capacity.

### 3.1. The Impact of Protein Oxidation on the Flavor of Dried and Fermented Meat Products

**Figure 3:**
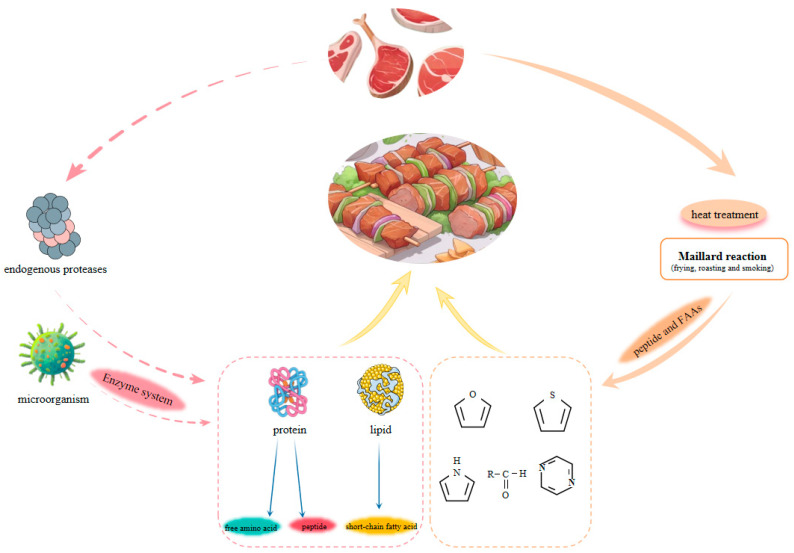
Flavor formation diagram of meat products.

In the process of manufacturing dry-cured meat items like ham and bacon, a sequence of chemical reactions takes place, which involves the oxidation modification of proteins. This process results in the generation of diverse amino acids and peptide compounds, playing a vital role in enhancing the flavor profile of dry-cured meat products. Moderate oxidation gives dry-cured meat its distinctive flavor and color, which is favored by consumers [49]. Excessive oxidation can cause increased protein breakdown and the production of an excessive quantity of small nitrogen compounds such as FAAs and peptides. This may negatively impact the flavor of dry-cured meat, resulting in the development of bitter or metallic tastes [50]. The oxidative degradation of dry-cured meat may occur due to enzymatic reactions caused by endogenous proteases and microbial activity. Endogenous proteases can be categorized as exopeptidases and endopeptidases. Exopeptidases mainly include aminopeptidases, while endopeptidases consist of calpain, cathepsin B, cathepsin D, and cathepsin L [51]. These endogenous enzymes break down myosin and myofibrillar proteins into peptides and FAAs (Figure 3) [52] and certain volatile flavor compounds, with aldehydes being the most prevalent during the storage of dry-cured ham [53]. Zhang et al. [54] analyzed the changes in protein hydrolysis products during the dry-curing of bacon. The findings indicated that protein degradation took place throughout the curing and aging stages, resulting in the creation of peptides, water-soluble nitrogen (WSN), and FAAs. Among them, peptides can directly promote flavor formation or indirectly become flavor precursors [55]. In their investigation on the impact of the Maillard reaction on the flavor of dry-cured meat products, Li et al. [56] discovered that FAAs resulting from protein degradation during the curing process actively engaged in the Maillard reaction, leading to the formation of volatile compounds (VCs). In addition, Schiff bases are also nitrogenous aromatic compounds. The Schiff base is the result of cross-linking between the carbonyl and amino groups of alkaline amino acids in meat proteins. Lorido et al. [57] conducted a study comparing frozen dry-cured meat to fresh meat in order to investigate the levels of protein oxidation, specifically Schiff base and carbonylation formation. The study findings showed a strong connection between the formation of Schiff bases and the flavors of saltiness and rancidity in dry-cured meat.

In conclusion, protein oxidation does not only have the positive contribution to flavor development in the production of dry-cured meat. Excessive protein oxidation can actually lead to undesirable flavors in dry-cured meat, impacting its overall quality. Therefore, to preserve the high-quality flavor of dry-cured meat products, a series of effective measures must be implemented to control and prevent excessive protein oxidation, thus avoiding adverse effects. Nitrite plays a crucial role in dry-cured meat products. It not only imparts the characteristic color to the meat and inhibits the growth of *Clostridium botulinum* but also enhances the flavor and provides antioxidant effects [58]. Higuero [59] conducted research on how decreasing the content of nitrite and nitrate in Iberian cured pork tenderloin impacts lipid and protein oxidation, as well as alterations in microbial composition. The results showed that compared with pork tenderloin prepared with 0, 37.5, and 70 mg/kg of NO_2_^−^/NO_3_^−^, the carbonyl content in the sample prepared with 150 mg/kg of NO_2_^−^/NO_3_^−^ was significantly reduced. This indicates that NO_2_^−^/NO_3_^−^ can effectively inhibit oxidation.

### 3.2. The Impact of Protein Oxidation on the Flavor of Roasted and Smoked Meat Products

Barbecuing is a popular way to prepare meat, creating a distinct aroma through chemical reactions between proteins and sugars, particularly the crucial “Maillard reaction.” This procedure produces advanced glycation end products (AGEs), including N^ε^-carboxyethyl lysine (CEL) and N^ε^-carboxymethyl lysine (CML), which serve as markers for the levels of AGEs present in various foods [60]. In research conducted by Li et al. [61], it was discovered that the total amount of lysine decreased in roasted beef in correlation with the dosage of NaCl and polyphosphate. Additionally, the protein carbonyl content in the roast beef patties showed a similar pattern to the reduction of tryptophan, which was dependent on the amount of salt used compared to the control group. These findings indicate that lysine acid residues in meat products are susceptible to oxidation or Maillard reactions when subjected to the process of roasting. Their oxidation leads to protein oxidation and an increase in protein carbonyls content. Through studying the impacts of diverse cooking methods on the oxidation of fish protein, Hu et al. [62] discovered that the process of baking and frying leads to a notable elevation in carbonyl and Schiff base levels, a decrease in free thiols content, and the formation of lipid-derived aldehydes such as 4-hydroxynonanal (HNE) and MDA. This interaction between these aldehydes can indirectly result in protein oxidation, with a more pronounced effect observed in baked and fried samples [63]. Throughout the heating procedure, a sequence of chemical reactions takes place. For instance, peptides, amino acids, nucleic acids, and bases are produced by the degradation of biological macromolecules. These substances are not only important sources of flavor and nutrition in meat products but also the main substrates involved in the Maillard reaction. The Maillard reaction results in the formation of a range of VCs, including pyrazines, aldehydes, pyrroles, ketones, furans, thiophenes, and others [64]. These VCs not only give meat products a distinctive aroma and flavor but also influence the color and taste of the meat products (Figure 3). Simultaneously, the oxidative deamination and decarboxylation of amino acids in the presence of α-dicarbonyl compounds generated during the Maillard reaction, resulting in the production of Strecker aldehydes. Strecker aldehydes are one of the main factors contributing to the distinct aroma of food. Hidalgo et al. [65] investigated an innovative mechanism for the synthesis of Strecker aldehydes, revealing that it originates from the breakdown of amino acids facilitated by the abundance of highly reactive carbonyl compounds. When amino acid-derived Strecker aldehydes are subjected to oxidative degradation, FAAs facilitate oxidative deamination and decarboxylation processes, leading to the generation of aromatic aldehydes like 3-methylbutyraldehyde (malt) or phenylacetaldehyde (honey-like). These substances play a substantial role in enhancing the overall aroma profile of food products [66]. Chao et al. [67] conducted an experiment to investigate the impact of varying roasting temperatures on protein oxidation and the modification of amino acid residue side chains in beef. Their findings revealed that elevated roasting temperatures led to increased levels of protein oxidation and modifications to amino acid residues, particularly aromatic amino acids, during the roasting process.

Smoking serves as a viable technique for food preservation, effectively prolonging the shelf life of various food items. The unique smoky taste found in smoked meat products is a result of the VCs produced when the smoked materials are heated and broken down [68]. Zhang et al. [69] also found that the content of heterocyclic aromatic amines (HAAs) increases with the prolongation of smoking time by comparing the formation of HAAs at diverse sugar fumigation durations. During the smoking process, the proteins in meat products undergo degradation to produce FAAs, which has a direct impact on the taste profile of the meat items. Merlo et al. [52] investigated the changes in FAAs and volatile organic compounds (VOCs) during bacon storage under 5 °C smoking conditions. They found that FAAs increased with prolonged storage time, which was mainly the result of protein hydrolysis. Guo et al. [70] conducted a study to examine the alterations in FAAs and volatile compounds in bacon subjected to various smoking techniques. Their findings indicated that liquid smoking can produce sensory attributes comparable to those achieved through conventional smoking methods. However, the traditional smoking method involves direct contact of wood-burning smoke with the product, which can cause the product to contain carcinogenic polycyclic aromatic hydrocarbons (PAHs) and pollute the environment. Therefore, liquid smoking, as a safe and environmentally friendly new method, is becoming increasingly popular. Soares et al. [71] studied the antioxidant and antibacterial properties of liquid smoke and its potential for use in bacon production. The results showed that liquid smoke has high inhibitory activity against *Staphylococcus aureus*, *Streptococcus cholerae*, *Listeria monocytogenes*, and *Escherichia coli*. Additionally, it has the ability to capture DPPH free radicals, which has antioxidant effects. At the same time, liquid smoking can effectively shorten the smoking time, thereby reducing costs.

Baked and smoked meat products typically generate a variety of flavor compounds as a result of the Maillard reaction, particularly pyrazines. Pyrazines are crucial flavor compounds in baked goods due to their typically robust baking aroma. Nevertheless, when FAAs engage in the Maillard reaction under elevated temperatures, they generate a range of heterocyclic volatile compounds, including but not limited to HAAs and PAHs. These compounds have the potential to influence the taste of meat products and present health hazards to consumers. PAHs may be regarded as potentially genotoxic and carcinogenic to humans. PAHs that consist of four fused rings, including benz[a]anthracene (BaA) and chrysene (CHR), exhibit weak carcinogenic properties. In contrast, PAHs with five or more fused rings, such as dibenz[a,h]anthracene (DhA), benzo[a]pyrene (BaP), indeno [1,2,3-cd]pyrene (IcP), benzo[b]fluoranthene (BbF), benzo[k]fluoranthene (BkF), and benzo[ghi]perylene (BgP), are considered to possess potential genotoxic and carcinogenic effects on humans [72]. HAAs are also a group of mutagenic substances that can be categorized into amino-imidazolines (AIA) and amino-carbolines (AC) [73].

### 3.3. The Impact of Protein Oxidation on the Flavor of Steamed Meat Products

Compared with raw meat, the total carbonyl content in steamed meat products is significantly increased, while the thiol content is reduced. The reason for this phenomenon is that the process of cooking alters the interactions between proteins, contributing to a gradual unfolding of the protein structure as the temperature rises [74]. Zhu et al. [75] studied the impact of cooking temperatures and durations on protein oxidation, protein degradation, and flavor formation of stewed chicken. They observed that SH groups and disulfide (S-S) bonds decreased with the increase in cooking durations. Additionally, they found that the temperatures of 80 and 90 °C, as well as cooking durations ranging from 50 to 60 min, were identified as pivotal factors influencing the SH/S-S exchange reaction. The significant presence of nonprotein nitrogen compounds (such as total volatile basic nitrogen, nitrogen found in FAAs, and peptides) and considerable protein breakdown in this context appear to play crucial roles in the development of flavor in chicken stew. Beyond this point, temperature and time do not significantly enhance flavor. Wang et al. [76] conducted a research investigation to assess the influence of diverse culinary methods on the volatile flavor compounds and non-volatile compounds found in Pingliang red beef. The findings indicated that the process of cooking Pingliang red beef resulted in the creation of a beef environment that exhibited increased hydrophilicity, which stimulated increased proteolysis and other aqueous processes. Proteolysis produces amino acids, peptides, and other small molecular compounds. These compounds are important components of beef flavor. Song [77] conducted a study that examined the impact of various cooking methods on the microstructure, quality, water distribution, and protein characteristics of meat. The study found that the myofibrillar structure of pork was disrupted during the cooking process, becoming more compact as the temperature increased. This change could potentially impact the binding ability of flavor substances with myofibrillar fibers [78,79]. Jin et al. [80] investigated the impact of diverse heating technologies on both non-volatile and volatile flavor components in salmon meat. The findings indicated that the steaming method resulted in a reduced presence of favorable flavor compounds in the salmon meat. Experiments have shown that during the cooking process of meat products, protein aggregation increases with the prolongation of boiling time, and aromatic compounds increase, but the denaturation of myofibrillar proteins is less [81]. Wang et al. [82] found that as the cooking time extended, proteins were gradually degraded, resulting in the production of more flavor precursors such as nucleotides and FAAs. FAAs are the primary outcomes of protein breakdown, and their levels and composition can impact the sensory attributes of meat products. The distinctive flavor of foods can be attributed to the interplay between various FAAs as well as their interactions with other flavor compounds. Yang et al. [83] conducted an analysis of adenosine triphosphate (ATP)-related compounds and FAAs in order to investigate alterations in water-soluble flavor compounds present in steamed grass fish. The researchers illustrated that ATP underwent degradation into adenosine diphosphate (ADP), adenosine monophosphate (AMP), and inosine monophosphate (IMP) as a result of steeping. The content of inosine 5′-IMP as the primary umami component in fish and shellfish was the highest. The content of threonine, serine, glycine, alanine, and proline with a fresh, sweet taste and lysine, histidine, and arginine with a slightly bitter taste in grass fish is higher. The effects of different steeping times on various FAAs are varied.

## 4. Technical Strategy

In recent times, there have been significant advancements in strategies and approaches for managing protein oxidation in meat products. Protein oxidation impacts the texture, taste, and nutritional content of meat products and can also reduce food quality and shelf life. Therefore, it is crucial to explore effective ways to manage protein oxidation in meat products. Adding antioxidants is a commonly used method to prevent protein oxidation. Physical methods can also hinder protein oxidation by changing the structure or surroundings of meat products. Biological approaches involve using bioactive substances like microorganisms or enzymes to regulate protein oxidation in meat products.

### 4.1. Add Antioxidants

At present, the traditional synthetic antioxidants commonly used in the meat processing industry are facing concerns regarding chemical toxicity and do not meet consumer demands. Therefore, it is an inevitable trend for natural antioxidants to replace synthetic antioxidants in food processing (Table 3). Plant polyphenols (like gallic acid, anthocyanins, catechins, ellagic acid, quercetin, arbutin, and so on) are among the most effective natural antioxidants studied in recent years. This primarily arises from the capacity of the single electron on the newly formed radical oxygen atom of phenolic antioxidants to engage in conjugation with the π electron cloud located on the benzene ring, which helps stabilize it [84]. When a tert-butyl group is located in the ortho position relative to the phenolic hydroxyl group, the presence of steric hindrance impedes the oxygen molecules’ attack. Consequently, the tert-butyl group diminishes the likelihood of alkoxy radicals initiating radical chain reactions, thereby demonstrating an antioxidant influence [85,86]. Li et al. [87] extracted total flavonoids (TFs) from Gnaphalium affine using pressure microwave assistance and evaluated their antioxidant activities in vitro. The results showed that the TFs exhibited significant DPPH, superoxide anion, and ABTS free radical scavenging activities, as well as ferric iron reduction/antioxidant properties and reducing ability. The variable is positively correlated with an increase in concentration. Deng et al. [88] examined how tea polyphenols, apple polyphenols, and cinnamon polyphenols impact the physical and chemical characteristics of smoked bacon. The study findings demonstrated that polyphenols were successful in reducing TBARS levels and carbonyl production, as well as in retarding sulfhydryl depletion in desiccated pork slices. This suggests that phenolic compounds play a role in inhibiting the oxidation of lipids and proteins. Gao et al. [89] conducted experiments to assess the antioxidant properties of theaflavins (TFs) and tea polyphenols (TP) in pickled sausage. Their findings showed a direct correlation between the inclusion of TFs and TP and their ability to scavenge DPPH free radicals when the quantities added ranged from 0.2 to 1 mg. The results indicated that TFs had a stronger antioxidant capacity compared to TP, indicating that TFs may have higher antioxidant potential than TP.

Carotenoids, one of the most widespread pigments in nature, are potent antioxidants in addition to plant polyphenols. The singlet oxygen in the excited state transfers energy to the carotenoid, leading to the transition of the carotenoid from its ground state to an excited state, which can then revert directly to the ground state [115]. Hamdi et al. [90] investigated the effect of turkey sausage on the quality characteristics of turkey sausage with turnip protein extract (CPE) extracted from crab shells. The data showed that the DPPH antioxidant capacity of CPE in the intestinal tissue of turkey meat exhibited a time-dependent pattern, with the scavenging activity against free radicals showing an augmentation over the duration of storage. CPE was effective in inhibiting the oxidation of myoglobin in turkey intestines.

In addition to preventing the oxidation of meat products, these additives can also enhance the safety and overall quality of meat processing. Antioxidants help preserve the flavor characteristics of meat products by interacting with proteins, decreasing protein oxidation, and safeguarding the structure and functionality of proteins. Sohaib et al. [91] studied the oxidative stability and flavor volatiles of chicken pâté throughout storage by treating it with antioxidants. The findings indicated that the inclusion of α-tocopherol and quercetin led to a notable decrease in the generation of undesirable aroma compounds in chicken pâté. These volatiles include aldehydes, ketones, hydrocarbon alcohols, and sulfur compounds. They are generated as a result of the degradation of fatty acids and amino acids.

In conclusion, antioxidants and their effects on meat products are very complex and diverse. From the capture of raw meat to its gourmet preparation, a variety of oxidative stress processes are involved. Therefore, effective and healthy antioxidants can reduce meat spoilage and minimize risks for consumers.

### 4.2. Physical Technology

Physical and technical treatments in meat processing and preservation can effectively inhibit protein oxidation by modifying the microstructure or environment of meat products. This method of inhibiting protein oxidation is crucial for preserving the quality of meat items and extending their shelf life. Packaging technology has been extensively employed in the industrial sector for the storage and preservation of meat products. Packaging creates a low-oxygen or even oxygen-free environment that significantly reduces the contact between meat products and oxygen, as well as the growth and reproduction of microorganisms. This process extends the shelf life of meat products (Table 3). Maqsood et al. [92] examined how different packaging methods affected the characteristics of camel meat. Their findings revealed that significant deterioration occurred in the wrapped samples after 14 days of storage, with air-packaged samples showing minimal degradation. In contrast, the protein fractions of vacuum-packed samples remained stable, suggesting that vacuum packaging was more successful in preventing oxidation. Li et al. [93] explored the effects of different oxygen concentrations (40%, 60%, and 80%) in modified atmosphere packaging (MAP) and vacuum packaging on the color and protein oxidation of pork under refrigeration. The results showed that both vacuum packaging and MAP packaging produced carbonyl groups during storage. However, the carbonyl content was higher in MAP packaging at a 60% oxygen concentration. The results above indicate that packaging technology alone cannot completely prevent protein oxidation in meat products. Therefore, auxiliary techniques such as irradiation, microwave treatment, and temperature regulation are necessary to achieve more effective antioxidant effects. The above results indicate that traditional packaging technology alone cannot completely prevent protein oxidation in meat products, so auxiliary irradiation and microwave technology are needed to achieve better antioxidant effects. According to Johnson et al. [94], the type of packaging can also affect the quality of meat products; the high-oxygen (80% O_2_)-modified atmosphere packaging (HIOX) can result in firmer meat products, and there are variations in protein degradation compared to vacuum packaging and polyvinyl chloride packaging. HIOX results in lower beef flavor and umami scores, along with an increased production of undesirable flavors such as oxidation, cardboard, and sourness. Various packaging types result in distinct protein degradation and the production of flavor compounds, which can influence the ultimate flavor and tenderness of the meat product.

Active packaging represents an emerging form of packaging technology that can prepare active packaging materials by fixing active agents on the surface of bulk packaging materials by ultraviolet radiation and plasma [116]. Fallah et al. [95] reviewed the combined effects of irradiation on various active packaged meat products. The analysis results revealed a significant 10.1% reduction in protein carbonyl levels in irradiated samples compared to non-irradiated biopolymer packaged and active packaged meat products. Irradiation also inhibits protein oxidation in meat products by damaging microbial cells and preventing the generation of ROS during their metabolism. Ham et al. [96] studied the effects of irradiation sources (X-rays, gamma rays, and electron beams) and dose levels on cooked beef patty and pork sausage. The study revealed that irradiation had a notable inhibitory effect on the proliferation of overall aerobic bacteria present in meat products, prevented the production of ROS during their metabolism, and thus inhibited protein oxidation in meat products. Irradiation can also decrease the incidence of fat oxidation in meat products, indirectly lowering the likelihood of protein oxidation. Teets et al. [97] studied the antioxidant properties of electron beam-irradiated almond skin powder on ground chicken breast throughout periods of cold storage and freezing. They found that the addition of almond skin powder could reduce the formation of peroxides under 20 and 30 kGy irradiation. The TBARS value inhibition rate of irradiated almond skin powder increased, indicating that the occurrence of lipid oxidation was inhibited during the experimental process. This indirectly reduces the possibility of protein oxidation. However, irradiation may cause the so-called “irradiation odor” and reduce the flavor of meat products, which limits the widespread adoption of irradiation technology in meat products. Huang et al. [98] studied the effects of gamma irradiation on the quality of smoked chicken breast meat. The researchers investigated the effects on taste and sensory attributes. The findings suggested that irradiation resulted in a rise in the overall concentration of unbound amino acids in smoked chicken breast meat. A dose of 3 kGy or higher was found to reduce protein oxidation levels. Lower doses of radiation were effective in enhancing the fresh amino acids while decreasing the levels of bitter and sweet-tasting amino acids. Upon irradiation at 6 kGy, the smoked meat emitted a sulfur smell, which is typically linked to the decomposition of amino acids and the cleavage of side chains. Ahn et al. [99] utilized sulfur-containing amino acids (Met and Cys) to investigate the mechanism of the odor generated by irradiation in meat. The results showed that sulfur-containing amino acid side chains were significantly vulnerable to erosion caused by irradiation, resulting in the formation of diverse sulfur compounds. These compounds were considered to be contributors to the odor of irradiated meat products. Wang et al. [100] studied the mechanism of the formation of irradiated beef odor based on metabolomics and found that the concentrations of cysteine and methionine decreased with the increase in irradiation dose. This decrease may be attributed to the susceptibility of these two sulfur-containing amino acids to attack by irradiation-free radicals, resulting in secondary oxidation. Therefore, cysteine and methionine metabolism may play a key role in the development of irradiated beef odor.

In addition to the aforementioned methods, microwave heating can effectively prevent protein oxidation in meat processing and preservation (Table 3). Taşkıran et al. [101] compared the effects of microwave heating and traditional oven cooking on the protein changes in chicken meat. The results showed that both microwave heating and traditional oven cooking could lead to the degradation of myofibrillar protein, but the protein degradation was lower under microwave heating. Dong et al. [102] studied the changes in the total antioxidant capacity of proteins in Penaeus South America under microwave conditions. The data indicated that as the microwave processing time and temperature increased, the total antioxidant capacity (TAC) of the sample also increased. Han et al. [103] investigated the effects of microwave and water bath heating on the structural changes of beef myofibrillar proteins. They also explored how these heating methods affect the interaction of keto flavor compounds with beef myofibrillar proteins. The findings indicate that microwave heating exerts a notable impact on protein structure through both thermal and non-thermal mechanisms. These alterations, particularly involving ketones, can impact the adsorption and release of flavor compounds in meat, consequently influencing the overall flavor characteristics profile of meat products. The impact of microwave heating on meat products may lead to significant changes in protein structure due to non-thermal effects.

It is important to acknowledge that while these physical techniques can partially impede protein oxidation in meat items, they do not entirely arrest the oxidation mechanism. Additionally, the oxidative resilience of meat products is influenced by various elements, including the quality of raw materials, processing parameters, and storage conditions. Consequently, a range of strategies must be implemented during the manufacturing and storage phases of meat products to manage protein oxidation, taking into account multiple factors.

### 4.3. Biotechnology

Biological approaches can regulate protein oxidation in meat products by utilizing bioactive substances such as microorganisms or enzymes (Table 3). Lactic acid bacteria (LAB) are common fermentative microorganisms. They can produce lactic acid and other organic acids to reduce the pH value of meat products. Additionally, they can scavenge DPPH and ABTS free radicals to alleviate oxidative stress, thereby inhibiting the activity of oxidases and the growth of harmful microorganisms and slowing down the oxidation rate of proteins [117,118]. Wang et al. [104] reviewed the regulatory role of LAB fermentation in the processing and storage of meat products in their article on the changes of proteins during the processing and storage of fermented meat products, as well as the regulation of LAB in these products. In the context of protein oxidation, microbial transaminase can convert FAAs, which are taste substances produced by protein degradation and oxidation, into α-keto acids and ultimately into the respective alcohols and acids. In addition, LAB, as a natural antioxidant, can prevent the overoxidation of proteins, which results in changes in protein conformation. Stadnik et al. [105] analyzed the effect of fermentation inoculated with LAB on the oxidation changes of dry-cured meat products. The findings indicated that following a 6-month fermentation period, the surface hydrophobicity of LAB samples exhibited a reduction in comparison to the control group that underwent spontaneous fermentation. This decrease resulted in a reduction in the quantity of sites capable of binding with the flavor compounds during the protein interaction, consequently impacting the protein’s capacity to effectively bind these flavor compounds. Lin et al. [106] investigated the effect of *Lactobacillus plantarum* on the flavor and oxidation of Chinese sausage. The research demonstrated that *Lactobacillus plantarum* P3-M2 and P3 possess significant antioxidant capabilities and contribute to improving the flavor of sausages through the mitigation of lipid and protein oxidation processes. Hu et al. [107] improved the taste of low-salt dried sausages by inoculating different LAB (*L. sakei*, *L. curvatus*, *W. hellenica*, and *L. plantarum*). The results showed that flavor substances, such as FAAs and organic acids, increased in the samples inoculated with LAB. Wen et al. [108] studied the effect of LAB (*P. acidilactici* BP2, *L. sakei* BL6, and *L. fermentum* BL11) on lipid oxidation and protein oxidation of beef jerky. Results show that the inoculation of glucosinolates, CHC, barbituric acid, and carbonyl content of reactants were significantly lower than the control group (*p* < 0.05). These findings suggest that LAB fermentation effectively inhibits lipid oxidation and protein oxidation. Han et al. [109] studied LAB isolated from Harbin sausage (*L. fermentum* R6, *P. pentosaceus* R1, *L. longer* R4, and *L. curvatus* R5, etc.) with probiotic properties. The data showed that different LAB strains exhibited varying degrees of ability to inhibit lipid peroxidation and demonstrated reducing power. This indicates that antioxidant activity is one of the most significant functions of LAB.

Some yeast can also generate antioxidants in meat products, such as GSH. These substances can react with oxidation products, reducing the occurrence of protein oxidation. Hou et al. [110] investigated the effects of selenium-rich *Saccharomyces cerevisiae* (SSC) and *Saccharomyces cerevisiae* (SC) on meat quality. They found that the experimental group could enhance the activities of thioredoxin reductase (TR) and glutathione peroxidase (GPx), as well as total antioxidant capacity. SSC exhibited more significant improvements. Thus, through stimulation of the glutathione and thioredoxin systems to enhance meat quality and oxidation stability, the addition of selenium can have a beneficial effect.

Proteases can also hydrolyze muscle proteins, altering the structure and characteristics of the protein, thereby affecting its sensitivity to oxidation. Moderate protease treatment can enhance the tenderness and flavor of meat products while decreasing the likelihood of protein oxidation. Broncano et al. [111] utilized various proteases (fungal protease, fungal protease concentrate, and neutral bacterial protease) to enhance the oxidative stability of fermented sausages. The study evaluated the antioxidant capabilities of three distinct proteases present in meat extracts through the assessment of four methodologies: free radical scavenging activity, metal chelation assay, reducing power, and inhibition of autooxidation of linoleic acid. The results showed that the addition of protease to sausage increased the antioxidant activity of sausage extracts, as well as the metal chelation assay. While other enzymes, such as catalase (CAT) and superoxide dismutase (SOD), can directly remove the superoxide anion in meat products and hydrogen peroxide, inhibiting the oxidation of proteins. Ahn et al. [112] added xanthine oxidase (XOD), SOD, and catalase to meat homogenate. The analysis results indicated that adding SOD and CAT alone or in combination could reduce the antioxidant capacity of chicken meat. Zhao et al. [113] studied the effect of proteasome treatment on volatile compounds and odor changes in the longissimus muscle of cattle. The results indicated that proteinase K treatment produced peptides with high Q values, leading to an enhancement in the average bitter taste of the identified peptides. On the other hand, the application of flavor enzymes and bromelain resulted in a notable elevation in ketones and odors, while excessive proteolysis induced by papain and proteinase K led to a substantial decrease in esters and aldehyde-like compounds. The level and degree of hydrolysis of amino acids were found to be the main factors regulating volatile compounds and odor levels. Hence, the utilization of enzymatic treatment plays a crucial role in influencing the taste profile of meat items. Dou et al. [114] examined the implications of alterations in oxidative stability on the overall quality and sensory characteristics of lamb meat derived from the biceps femoris (BF) and longissimus dorsi (LD) muscles in lambs during the postmortem aging process. They showed that the activities of SOD, CAT, and glutathione peroxidase (GSH-Px) gradually decreased over time during postmaturation. Reduced activity of these enzymes may lead to increased protein oxidation reactions in meat products, which, in turn, may affect the flavor of the meat products.

In meat processing, both microorganisms and enzymes can be utilized to control protein oxidation. For instance, in fermented meat products, organic acids produced by microorganisms like LAB can lower the pH value. Additionally, the inclusion of proteases can enhance the tenderness and flavor of meat products. The combined effect of these two factors can be significant.

## 5. Outlook and Summary

The oxidation of proteins produces a series of flavor precursors that, through subsequent chemical reactions, form various volatile flavor compounds that significantly contribute to the overall flavor of meat products. Moderate protein oxidation helps improve the flavor quality of meat products, resulting in a richer aroma and better taste. Excessive oxidation, however, can lead to the production of undesirable flavors such as bitterness, sourness, and off-flavors. Protein oxidation has varying effects on flavor in different meat products. For example, in dry-cured fermented meat, protein oxidation primarily breaks down into flavor precursors, thus influencing the flavor. The FAAs resulting from protein oxidation participate in the Maillard reaction, forming a variety of volatile flavor compounds in grilled and smoked meat products. During the cooking process, water-soluble proteins in meat undergo significant degradation, leading to the destruction of the protein structure. This, in turn, impacts the interaction between proteins and flavor compounds.

In recent years, some progress has been made in understanding the mechanisms by which protein oxidation affects the flavor of meat products. However, protein oxidation still needs to be addressed in conjunction with quality control and safety considerations during actual production. There are various methods and techniques for regulating protein oxidation in meat products, each with its own unique advantages and limitations. In practical applications, suitable control methods can be chosen based on the types of meat products, processing techniques, and quality standards to attain optimal outcomes. In the production process, it is crucial to effectively control the oxidation of myofibrillar proteins and to detect and manage the degree of oxidation to ensure the quality and safety of meat products. Future research should focus on addressing these problems in actual production settings to offer improved technical assistance for the advancement of the meat industry.

In summary, there are still many issues that need to be explored and solved in the study of the mechanism of the impact of protein oxidation on the flavor of meat products. Future research should focus on gaining a deeper understanding of the chemical changes during oxidation, exploring their impact mechanisms on flavor, developing effective antioxidant technologies, and applying research findings to practical production. These studies will help us better understand and control the quality and flavor of meat products and promote the development of the meat industry.

## Figures and Tables

**Table 1:** Possible mechanisms of lipid oxidation induced protein oxidation.

Stage	Reaction	Formula
Initiation	L → L·	(e)
Transmission	L· + O_2_ → LOO·	(f)
Hydrogen abstraction	LOO· + P → LOOH+P·(-H)	(g)
Prolongation	LOO·+ P → ·LOOP	(h)
Composition	·LOOP + P + O_2_ → ·POOLOOP	(i)
Polymerization	P-P· + P· + P → P-P-P· + P-P-P	(j)

Note: L = lipid; L**·** = lipid radical; LOO**·** = lipid peroxy radical; LOOH = lipid alkyl peroxide; P**·**(-H) = protein alkoxy radical; **·**LOOP = cross-linked derivative; **·**POOLOOP = protein–lipid polymers; P-P**·,** P-P-P**·**, and P-P-P = polymers.

**Table 2:** The side chains of amino acid residues and their corresponding oxidation products.

Amino Acid	Products of Oxidation
Arg	Glutamate semialdehyde
Cys	-S-S-, -SOH, -SOOH
His	2-oxo-histidine, 4-OH-glutamic acid, aspartic acid, aspartyl ammonia
Leu	3-, 4-, and 5-OH-leucine
Met	Methionine sulfoxide
Phe	2, 3-dihydroxyphenylalanine, 2-,3-, and 4-hydroxyphenylalanine
Tyr	3, 4-dihydroxyphenylalanine, 3-nitrotyrosine, tyrosine–tyrosine cross-linking
Try	2-, 4-, 5-, 6-, and 7- hydroxytryptophan, 3-kynurenine, nitro, formylkynurenine,
Thr	Try
Pro	2-amino-3-keto-butyric acid
Glu	Glutamyl semialdehyde, 2-pyrrolidine, 4- and 5-O-proline, pyroglutamic acid.
Lys	Oxalic acid, pyruvate complex, α-aminoadioyl semialdehyde

**Table 3:** Examples of regulatory technical strategies for mitigating protein oxidation in meat products.

Technology Strategy	Details	Objects	Sample	References
Antioxidants	Gnaphalium affine	Liver of mice	Good in vivo and in vitro antioxidant capacity.	[87]
	Tea polyphenol, apple polyphenol, and cinnamon polyphenol	Dry-fried bacon	Reduces protein oxidation and enhances bacon safety.	[88]
	Theaflavins and tea polyphenols	Cured sausage	Inhibit the oxidation of ferromyoglobin to metmyoglobin in sausage.	[89]
	Carotenoproteins extract	Turkey meat; sausages	Enhancing the antioxidant and antibacterial properties of meat products.	[90]
	Quercetin dihydrate and α-tocopherol	Chicken meat patties	Improve the oxidation resistance, storage, and quality of cooked meat pies.	[91]
Physical technologies	different packaging conditions (air, vacuum wrapped, modified atmosphere, and so on)	Camel meat; pork; beef strip loins	Different packaging types result in varying degrees of protein degradation and the formation of flavor compounds.	[92] [93] [94]
	Combination of ionizing radiation and bio-based active packaging	Muscle foods	Lipid and protein oxidation is diminished, and the growth rate of the microbiota is decreased.	[95]
	Different sources of irradiation (gamma rays, electron beams, and X-rays)	Beef patties and pork sausages; chicken meat; smoked chicken breast; sulfur-containing amino acid monomers; yak meat	Inhibit the growth of aerobic bacteria and the oxidation of meat proteins; the metabolism of cysteine and methionine may play a crucial role in the development of irradiation odor.	[96] [97] [98] [99] [100]
	Microwave cooking	Chicken meat; shrimp; fresh beef samples	Effectively inhibit protein oxidation; affects the flavor of meat products by affecting the protein structure.	[101] [102] [103]
Biotechnology	LAB (*L. plantarum*; *L. curvatus*; *L. sakei*; *W. hellenica*; *P. acidilactici BP2*; *L. fermentum BL11*; *P. pentosaceus R1*, *L. brevis R4*, *L. curvatus R5*, *and L. fermentum R6*)	Fermented meat products; pork loins; sausages; beef jerky; Harbin dry sausages	Effectively inhibits oxidation of meat products and improves flavor.	[104] [105] [106] [107] [108] [109]
	*S* *accharomyces cerevisiae and selenium-enriched Saccharomyces cerevisiae*	Broiler chickens	Effectively improve meat quality and oxidation stability.	[110]
	Neutral bacterial protease, fungal protease, and fungal protease concentrate	Fermented sausages	*Effectively inhibit protein oxidation.*	[111]
	Xanthine oxidase, superoxide dismutase, and catalase	Raw turkey meat	*Showed lower antioxidant capacity.*	[112]
	Enzymes proteinase K, papain, bromelain, and Flavourzyme	Beef longissimus dorsi	Enzymatic treatment has a great influence on the flavor of meat products.	[113]
	Superoxide dismutase, catalase, and glutathione peroxidase	Biceps femoris and longissimus dorsi muscles of lambs	The decrease in enzyme activity may lead to an increase in protein oxidation reactions in meat products, thereby affecting their flavor.	[114]

## Data Availability

No new data were created or analyzed in this study. Data sharing is not applicable to this article.
